# Supplementary material for: Sero-epidemiological survey of Coxiella burnetii in livestock and humans in Tana River and Garissa counties in Kenya
Source: PLoS Negl Trop Dis. 2022 Mar 3;16(3):e0010214. doi: 10.1371/journal.pntd.0010214 (PMC8923444; doi:10.1371/journal.pntd.0010214)
Supplement: S1 Table — (DOCX) [file pntd.0010214.s004.docx]

**S1 Table: AIC values for all the multivariable models used to analyse livestock and human data, together with the independent variables included in each model.**

| **Multivariable models used to analyse livestock data** | | |
| --- | --- | --- |
| **Model** | **AIC** | **Independent variables included in the model** |
| Model 1 (null model) | 1952.8 | Intercept-only model without explanatory variables. |
| Model 2 (maximal model) | 1834.5 | Species (cattle, goats, sheep), animal sex (male, female), sampling area (Bura, Hola), age (calf/kid/lamb, weaner, adult), land use type (pastoral, irrigation). |
| Model 3 | 1832.6 | Species (cattle, goats, sheep), animal sex (male, female), sampling area (Bura, Hola), age (calf/kid/lamb, weaner, adult). |
| Model 4 (final model) | 1831.0 | Species (cattle, goats, sheep), animal sex (male, female), age (calf/kid/lamb, weaner, adult). |
| **Multivariable models used to analyse human data** | | |
| Model 1 (null model) | 1086.6 | Intercept-only model without explanatory variables. |

AIC, Akaike’s Information Criteria.
