# Supplementary material for: Sero-epidemiological survey of Coxiella burnetii in livestock and humans in Tana River and Garissa counties in Kenya
Source: PLoS Negl Trop Dis. 2022 Mar 3;16(3):e0010214. doi: 10.1371/journal.pntd.0010214 (PMC8923444; doi:10.1371/journal.pntd.0010214)
Supplement: Attachment — Submitted filename: Response to editorial comments.docx [file pntd.0010214.s005.docx]

**Comment:** We note that the following authors have not yet confirmed their authorship on this manuscript:

    Damaris Mwololo
    Philip Kitala
    Joan Karanja
    Rosemary Sang
    Delia Grace
    Bernard Bett
At this point this request is not urgent; we have re-sent their co-author confirmation email containing an auto-confirmation link for them to click, and we will reach out to them at a later stage if we don't hear from them. We wanted to mention this in case you are aware of them experiencing technical issues with confirming authorship via the link. If so, please do ask them to contact us directly via email and we'd be happy to assist.

**Response:** Thank you for this information. I have requested all the authors to confirm their authorship in this manuscript.

**Comment:** Please ensure that the CRediT author contributions listed for every co-author are completed accurately and in full.
At this stage "all Authors" require contributions.  Please ensure that the full contributions of each author are acknowledged in the ""Add/Edit/Remove Authors"" section of our submission form.
**Response:** We have ensured that the full contributions of each author is acknowledged in the submission form.

**Comment:** Please provide separate figure files in .tif or .eps format. You do not need to remove any figures embedded in your manuscript file, so long as you have provided separate figure files.  Please ensure that all files are under our size limit of 20MB.

For more information about figure files please see our guidelines:
<https://journals.plos.org/plosntds/s/figures>
<https://journals.plos.org/plosntds/s/figures#loc-file-requirements>
Please provide us with a direct link to the base layer of the map used in "Figure 1" and ensure this location is also included in the figure legend.

**Response:** We have provided a separate figure in .tif and within the required size of less than 20MB. The base layer of the map used to create Figure 1 was included in the figure legend. We also provided a direct link to where this base layer was downloaded (<https://www.diva-gis.org/gdata>) (page 36, lines 833-834 in the manuscript with tracked changes).

**Comment:** We have noticed that you have uploaded supplementary table 1 as supporting information but you have not included them in the list of legends.  Please add a citation of this SI data files into the list of legends of your supporting information files.

**Response:** We have included supplementary table 1 in the list of legends (page 33, line 784 in the clean copy of the manuscript and page 34, line 813 in the manuscript copy with tracked change.

**Comment:** Please ensure that the funders and grant numbers match between the Financial Disclosure field and the Funding Information tab in your submission form. Note that the funders must be provided in the same order in both places as well. Currently, Department for International Development (DFID), the Economic and Social Research Council (ESRC) and the Natural Environment Research Council (NERC) are missing from the Funding Information.
**Response**: Under financial disclosure field, we noted that a statement of funding sources is required which will be included in the manuscript if published. The following statement with the names of funders and grant numbers was therefore provided. ‘‘The sampling of livestock and humans was implemented under the project: Dynamic Drivers of Disease in Africa: Ecosystems, livestock/wildlife, health and wellbeing (grant no. NEJ001570), funded by the Ecosystem Services for Poverty Alleviation, Programme (ESPA). The ESPA programme was funded by the Department for International Development (DFID), the Economic and Social Research Council (ESRC) and the Natural Environment Research Council (NERC). Additional funding for data analysis and manuscript development was provided through Co-infection with Rift Valley fever virus, Brucella spp. and Coxiella burnetii in humans and animals in Kenya: Disease burden and ecological factors (Co-infection project) funded by the Defense Threat Reduction Agency, contract number HDTRA 11910031. The funders had no role in study design, data collection and analysis, decision to publish, or preparation of the manuscript’’.

However, since under funding information tab specific information on the funding sources was required, we have also indicated that funding for this study was provided by Ecosystem Services for Poverty Alleviation, Programme (ESPA) and Defense Threat Reduction Agency. These two funders were listed in the same order as they appear under financial disclosure section. The grant numbers and the names of the recipients of these grants were also provided.

**Comment:** In the online submission form, you indicated that you need assistance with your Data Availability Statement. Please reply to us via return email so we can best address your concerns and remedy your Data Availability Statement. If the box for this was ticked in error, please update your submission form answer, and ensure your Data Availability Statement is compliant. All PLOS journals require data underlying the findings described in the manuscript to be freely available to other researchers, either in a public repository, or in the manuscript itself. This policy applies to all data except where public deposition would breach compliance with the protocol approved by your research ethics board. If your data cannot be made publicly available for ethical or legal reasons (e.g., public availability would compromise patient privacy), please explain your reasons by return email and your exemption request will be escalated to the editor for approval. Your exemption request will
be handled independently and will not hold up the peer review process, but will need to be resolved should your manuscript be accepted for publication. One of the Editorial team will be in touch if they require more information.

**Response:** The box on data availability statement was ticked by mistake. We have unticked this box since we had already indicated that all data used in this manuscript are available without restrictions. Besides, all data used in this manuscript were provided as additional supplementary files. Data availability statement had also provided in the revised manuscript and it reads ‘‘All relevant data are within the paper and its supporting information files’’ page 24-25, lines 588-589).
