# Supplementary material for: Sero-epidemiological survey of Coxiella burnetii in livestock and humans in Tana River and Garissa counties in Kenya
Source: PLoS Negl Trop Dis. 2022 Mar 3;16(3):e0010214. doi: 10.1371/journal.pntd.0010214 (PMC8923444; doi:10.1371/journal.pntd.0010214)
Supplement: Attachment — Submitted filename: Response to editorial comments - R2.docx [file pntd.0010214.s006.docx]

**Comment**. We note that the following authors have not yet confirmed their authorship on this manuscript: Philip Kitala, Delia Grace
At this point this request is not urgent; we have re-sent their co-author confirmation email containing an auto-confirmation link for them to click, and we will reach out to them at a later stage if we don't hear from them. We wanted to mention this in case you are aware of them experiencing technical issues with confirming authorship via the link. If so, please do ask them to contact us directly via email and we'd be happy to assist.

**Response:** Thank you for this information. I have requested all the authors to confirm their authorship in this manuscript.

**Comment**. When completing the data availability statement of the submission form, you indicated that  "All relevant data are within the manuscript and its Supporting Information files.". However, you also checked the following box: “Tick here if the URLs/accession numbers/DOIs will be available only after acceptance of the manuscript for publication so that we can ensure their inclusion before publication.” If this box was checked in error, and there is no additional data to deposit, please ensure this box is unticked when you resubmit.

**Response:** The box on data availability statement was ticked by mistake. We have unticked this box since we had already indicated that all data used in this manuscript are available without restrictions. Besides, all data used in this manuscript were provided as additional supplementary files. Data availability statement had also provided in the revised manuscript and it reads ‘‘All relevant data are within the paper and its supporting information files’’ page 25, lines 591-592).
Comment: Please amend your detailed Financial Disclosure statement. This is published with the article, therefore should be completed in full sentences and contain the exact wording you wish to be published. **Response**: We have provided a detailed financial disclosure statement which will be included in the manuscript if published. The following statement with the names of funders and grant numbers was provided. ‘‘The sampling of livestock and humans was implemented under the project: Dynamic Drivers of Disease in Africa: Ecosystems, livestock/wildlife, health and wellbeing (grant no. NEJ001570), funded by the Ecosystem Services for Poverty Alleviation, Programme (ESPA). The ESPA programme was funded by the Department for International Development (DFID), the Economic and Social Research Council (ESRC) and the Natural Environment Research Council (NERC). Additional funding for data analysis and manuscript development was provided through Co-infection with Rift Valley fever virus, Brucella spp. and Coxiella burnetii in humans and animals in Kenya: Disease burden and ecological factors (Co-infection project) funded by the Defense Threat Reduction Agency, contract number HDTRA 11910031. The funders had no role in study design, data collection and analysis, decision to publish, or preparation of the manuscript’’.

**Comment:** State the initials, alongside each funding source, of each author to receive each grant.

Response: Since under funding information tab specific information on the funding sources was required, we have also indicated that funding for this study was provided by Ecosystem Services for Poverty Alleviation, Programme (ESPA) and Defense Threat Reduction Agency. These two funders were listed in the same order as they appear under financial disclosure section. The grant numbers and the names of the recipients of these grants were also provided as shown in the below
